# The 3’ UTR polymorphisms rs3742330 in *DICER1* and rs10719 in *DROSHA* genes are not associated with primary open-angle and angle-closure glaucoma: As case-control study

**DOI:** 10.1371/journal.pone.0284852

**Published:** 2023-04-26

**Authors:** Altaf A. Kondkar, Taif A. Azad, Tahira Sultan, Essam A. Osman, Faisal A. Almobarak, Glenn P. Lobo, Saleh A. Al-Obeidan

**Affiliations:** 1 Department of Ophthalmology, College of Medicine, King Saud University, Riyadh, Saudi Arabia; 2 Glaucoma Research Chair in Ophthalmology, College of Medicine, King Saud University, Riyadh, Saudi Arabia; 3 King Saud University Medical City, King Saud University, Riyadh, Saudi Arabia; 4 Department of Ophthalmology and Visual Neurosciences, University of Minnesota, Minneapolis, MN, United States of America; University of Florida, UNITED STATES

## Abstract

**Aim:**

In a retrospective and exploratory case-control study, we examined the genetic association of two common polymorphisms in the 3’ untranslated region (UTR) of *DICER1* (rs3742330) and *DROSHA* (rs10719) genes in primary open-angle glaucoma (POAG) and primary angle-closure glaucoma (PACG), and its related clinical phenotypes in a Saudi cohort.

**Methods:**

DNA genotyping was performed using TaqMan real-time PCR assays in 500 participants, including 152 POAG, 102 PACG, and 246 non-glaucomatous controls. Statistical analyses were performed to examine the association(s).

**Results:**

Allele and genotype frequency of rs3742330 and rs10719 did not vary significantly in POAG and PACG compared to controls. No significant deviation was observed from Hardy-Weinberg Equilibrium (*p* > 0.05). Gender stratification revealed no significant allelic/genotype association with glaucoma types. Also, these polymorphisms showed no significant genotype effect on clinical markers such as intraocular pressure, cup/disc ratio, and the number of antiglaucoma medications. Logistic regression showed no effect of age, sex, rs3742330, and rs10719 genotypes on the risk of disease outcome. We also examined a combined allelic effect of rs3742330 (A>G) and rs10719 (A>G). However, none of the allelic combinations significantly affected POAG and PACG.

**Conclusions:**

The 3’ UTR polymorphisms rs3742330 and rs10719 of *DICER1* and *DROSHA* genes are not associated with POAG and PACG or its related glaucoma indices in this Middle-Eastern cohort of Saudi Arab ethnicity. However, there is a need to validate the results on a broader population and other ethnicities.

## Introduction

Glaucoma is a chronic neurodegenerative disease. It is characterized by high intraocular pressure (IOP), trabecular meshwork (TM) dysfunction, apoptosis of the retinal ganglion cells (RGC), damage to the optic nerve head, and progressive visual field loss leading to blindness [1]. The most common types of glaucoma are primary open-angle glaucoma (POAG) and primary angle-closure glaucoma (PACG). POAG is characterized by an open iridocorneal angle, and PACG has a narrow or closed iridocorneal angle that blocks the aqueous humor (AH) outflow pathway leading to high IOP and subsequent glaucoma features [1]. PACG is far more common among Asians, including Saudi Arabs [2,3]. Aging, high IOP, race, myopia, family history, and genetic factors are well-known glaucoma risk factors [4]. POAG and PACG commonly follow a genetically complex inheritance pattern wherein genes and environmental factors contribute significantly to disease pathogenesis [5]. Many genome-wide association studies have identified genes and loci associated with these glaucoma types [6–8]. However, most of these studies have been performed on Asians and Caucasians, with conflicting reports on other ethnicities, including Saudi Arabs [9–11]. The specific genetic factors and molecular mechanisms contributing to glaucomatous eye damage are still elusive.

POAG patients exhibit a wide variety of both ocular and non-ocular vascular abnormalities. These critical vascular abnormalities must be addressed to understand the underlying pathogenic mechanisms in RGC and optic nerve degeneration [12]. For example, common genetic variations in *NOS3* and the *CAV1/CAV2* genomic regions, which code for proteins involved in setting the vascular tone, are associated with POAG [13,14]. Thus the systemic involvement in POAG cannot be completely ruled out. Likewise, although the mechanism for PACG is primarily mechanical, the overlapping clinical features among different glaucoma types suggest a common down-stream mechanism(s) in the development and/or progression of these diseases. Genetic association studies have identified polymorphisms common between glaucoma types [15–18]. These mechanisms may not represent a unifying hypothesis in our understanding of POAG or PACG development, but they do appear to play an essential role in disease pathogenesis. As the genetic basis of POAG and PACG in the middle-eastern Saudi Arabs is still largely unknown, we explored the genetic association between two common polymorphisms, rs10719 (A>G) in *DROSHA1* and rs3742330 (A>G) in *DICER1* and POAG and PACG in this ethnic population.

DROSHA and DICER are two critical RNase III enzymes, involved in the biogenesis and regulation of microRNAs (miRNAs). DROSHA is involved in the cleaving of primary-miRNA into a 70-bp stem loop precursor-miRNA in the nucleus. Whereas, DICER cleaves the double-stranded precursor-miRNA, transported into the cytoplasm by exportin-5, to produce miRNA and small interfering RNA (siRNA) [19]. MiRNAs are small (~22bp), conserved, non-coding RNAs that bind to complementary sequences in the 3′ untranslated region (3’ UTR) of messenger RNAs (mRNAs) and regulate posttranscriptional gene expression by inducing translational inhibition [20]. Differential expression of miRNAs with diagnostic potential in glaucoma-affected tissues, such as AH, plasma, tears, TM, and retina, and the demonstrated essential role for miRNAs in IOP regulation, RGC survival, and optic nerve damage suggests their potential involvement in glaucoma pathogenesis [21–24].

Dysregulation of miRNA processing has been demonstrated to facilitate cellular transformation and tumor formation [25]. Polymorphisms rs10719 (A>G) in *DROSHA1* and rs3742330 (A>G) in *DICER1* are located in the 3’ UTR region of their respective genes. The region may be necessary for miRNA binding, transcription factor binding, DNA methylation, and histone modification, suggesting that these genes might have critical regulatory functions [26–28] (**S1 Fig**). Interestingly, these polymorphisms have been reported to result in aberrant expression of these enzymes [29–32] that may alter miRNA expression in the cells [33,34], thus affecting the expression of corresponding genes and thereby deregulating downstream mechanisms/pathways that control cellular functions and have pathological consequences [29,35,36] (**S2 Fig**). Moreover, these polymorphisms have also been previously associated with several complex human diseases. These include hypertension, endometriosis, cancer, atherosclerosis, Parkinson’s, and pseudoexfoliation glaucoma [37–43].

DICER and DROSHA are critical enzymes in miRNA biogenesis, but their association with POAG and PACG has not been investigated [19]. We have previously reported an association of rs3742330 in *DICER1* in pseudoexfoliation glaucoma [43]. We hypothesize that polymorphisms (rs10719 and rs3742330) in *DROSHA*/*DICER1* genes would alter these enzymes’ expression, affecting miRNA production or regulation which might affect downstream pathways related to glaucoma processes (e.g., extracellular matrix remodeling and trabecular meshwork homeostasis) and influence the disease development and progression. Thus, we investigated their genetic association in POAG and PACG patients of Saudi origin.

## Materials and methods

### Study design and participants

We performed a retrospective and exploratory case-control study. The study adhered to the guidelines of the Declaration of Helsinki for human research and was approved by the Institutional Review Board committee (IRB protocol number # 08–657) at the College of Medicine, King Saud University, Riyadh, Saudi Arabia. Written informed consent was obtained from all participants. Study participants were recruited at the King Abdulaziz University Hospital in Riyadh, Saudi Arabia. Trained glaucoma specialists carried out the patient phenotyping. In general, we adopt the European Glaucoma Society [44] guidelines for diagnosis in our facility. A standardized ophthalmic examination was performed on all the participating patients This included measurement of IOP by Goldmann applanation tonometry mounted at the slit lamp, examination of anterior chamber angles by gonioscopy, dilated pupil examination of the lens and fundus, and visual field testing by Humphrey automated field analyzer. POAG patients (n = 152) satisfied the following clinical criteria: (1) the presence of glaucomatous optic neuropathy (defined as loss of neuroretinal rim with a vertical cup-to-disc ratio of >0.7 or an inter-eye asymmetry of >0.2, and/or notching attributable to glaucoma); (2) and corresponding visual field (Humphrey Field Analyzer II, Carl Zeiss Meditec, Inc., Dublin, CA, USA; using a full threshold 24–2 program) abnormalities typical of glaucoma such as nasal step defect, arcuate or paracentral scotomata, or generalized tunnel vision; (3) bilaterally open anterior chamber angles by gonioscopy; (4) adult-onset of the disease; (5) IOP ≥21 mmHg in one or both eyes before initiation of glaucoma treatment; (6) and absence of secondary causes of glaucomatous optic neuropathy with identifiable causes such as exfoliative glaucoma; angle-closure; pigmentary glaucoma; post-traumatic, infectious or inflammatory glaucoma (e.g., uveitis), post-surgical and post-medication (after corticosteroids, for example) [45]. The angle-closure glaucoma participants included chronic PACG patients (n = 102) exhibiting clinical evidence of anatomically closed angle showing the occurrence of appositional or synechial closure of the anterior chamber angle (~270° of the angle is occluded); high IOP (≥21 mmHg); optic disk damage with cup/disc ratio of ~0.7 (at least in one eye); and evidence of peripheral or advanced visual field defect [46]. A representative image of visual field finding in POAG and PACG patients is shown in **S3 Fig**. Participants with secondary types of glaucoma cases such as pigmentary glaucoma, uveitic, pseudoexfoliation, history of optic neuropathies or visual impairment not related to glaucoma, use of steroids, ocular trauma, absence of sufficient fundus visualization for disk assessment, or refusal to participate were excluded from the study.

A group of healthy non-glaucomatous Saudi Arab participants (n = 246) recruited from our ophthalmology screening clinics were included as controls in the study. These participants were: >40 years of age, with normal IOP without medication (<21 mmHg), open angles on gonioscopy, healthy optic disc (cup/disc ratio <0.5), free from any form of glaucoma on examination, and no family history of glaucoma. Subjects refusing to participate in the study were excluded.

### Genotyping of rs3742330 in *DICER1* and rs10719 in *DROSHA*

DNA samples extracted from peripheral EDTA blood were genotyped for these polymorphisms as described earlier [43]. Commercially available TaqMan^®^ assays: C__27475447_10 and C___7761648_10 (Catalog number: 4351379, Applied Biosystems Inc., Foster City, CA, USA) were used for genotyping rs3742330 [A>G] and rs10719 [G>A], respectively under recommended amplification conditions on ABI 7500 Real-Time PCR System (Applied Biosystems). Each PCR reaction was performed in a total volume of 25 μL consisting of 1X TaqMan^®^ Genotyping Master Mix (Applied Biosystems), 1X SNP Genotyping Assay Mix, and 20 ng DNA. Each 96-well plate included two no-template (negative) controls. The fluorescence analysis used the automated 2-color allele discrimination software to identify the rs3742330 (*DICER1)* and rs10719 (*DROSHA)* genotypes on a two-dimensional graph.

### Statistics

The deviation from Hardy-Weinberg Equilibrium (HWE) and dichotomous variables such as gender distribution between cases and controls, allele and genotype frequency distribution between cases and controls were tested using Pearson’s Chi-square analysis and Fisher’s test where applicable. Normality testing of continuous variables was done using the Kolmogorov–Smirnov test. Accordingly, the Mann-Whitney U test was used for a two-group comparison and the Kruskal-Wallis test for a three-group comparison. Logistic regression analysis was performed to test the effects of multiple risk factors (age, sex, and genotypes) on disease outcomes. The analyses were performed using SPSS version 22 (IBM Inc., Chicago, Illinois, USA), Stat View software version 5.0 (SAS Institute, Cary, NC, USA), and SNPStats online software (https://www.snpstats.net/start.htm) [47]. The combined allelic effect was estimated using SHEsis (http://analysis.bio-x.cn/myAnalysis.php). Power analysis used an open-source online PS program version 3.1.2 for unmatched case-control (dichotomous) testing (https://vbiostatps.app.vumc.org/ps/). A *p* < 0.05 (two-tailed) was considered statistically significant. Bonferroni’s correction *p*-value for multiple testing was considered where applicable.

## Results

### Demographic characteristics of the participants

**Fig 1:**
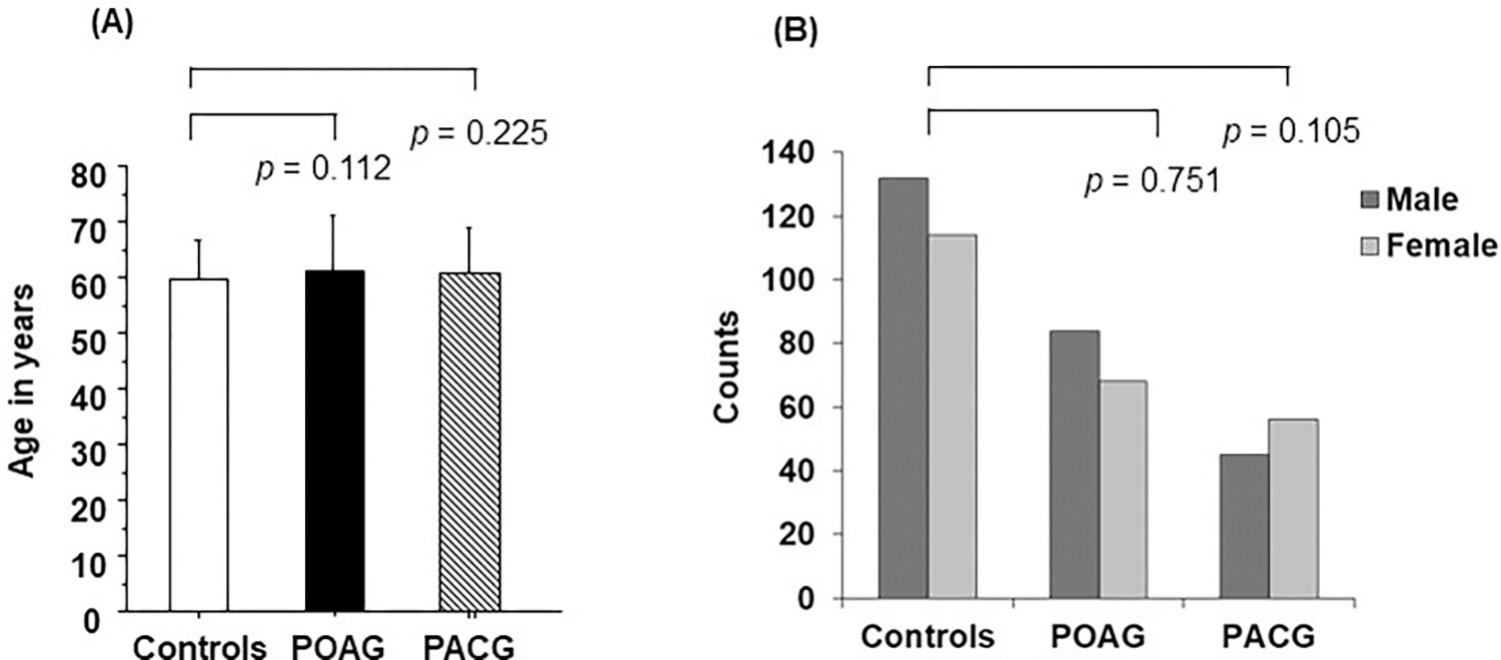
Demographic data of patients and controls. (A) The graph with error bars represents mean age and standard deviation with a p-value estimated by Mann-Whitney U-test compared to controls. (B) The gender distribution is represented as a frequency bar graph, and the p-value is calculated by Pearson’s Chi-square analysis.

The mean age of POAG patients was 60.9 y (± 10.4), PACG patients were 60.6 y (± 7.2), and controls were 59.5 y (± 7.2). The age differences were insignificant between POAG and controls (p = 0.112) and PACG and controls (p = 0.225). Similarly, there was no significant difference in gender distribution between POAG (84 men and 68 women) and PACG (45 men and 57 women) as compared to controls (132 men and 114 women). The demographic characteristics of participants are shown in **Fig 1**.

### Allele frequency distribution of rs3742330 (*DICER1*) and rs10719 (*DROSHA*)

Genotyping of rs3742330 and rs10719 was performed in 246 controls, 152 POAG, and 102 PACG DNA samples. Genotyping results were available for 241 controls, 152 POAG, and 102 PACG patients for *DICER1* rs3742330 polymorphism, and 246 controls, 150 POAG, and 101 PACG patients for *DROSHA* rs10719 polymorphism. These genotyping results were used to test for allele and genotype association between polymorphisms and POAG/PACG, and for clinical markers in the patient groups. However, eight samples missing either rs3742330 (n = 5 controls) or rs10719 (n = 2 POAG and n = 1 PACG) genotypes were excluded from regression and haplotype analysis.

The polymorphisms showed no significant deviation from HWE (*p* > 0.05). The rs3742330 [G] minor allele frequency (MAF) did not show any significant association with POAG (MAF = 0.08) and PACG (MAF = 0.07) as compared to the controls (MAF = 0.08). Furthermore, gender stratification also showed no significant association. Likewise, the MAF of rs10719 [A] also showed no association with POAG (MAF = 0.42) and PACG (MAF = 0.45) in comparison to controls (MAF = 0.43). Furthermore, both polymorphisms showed no gender-specific association with both glaucoma types compared to controls. The overall and gender-stratified MAF distribution of rs3742330 and rs10719 in cases and controls are shown in **Table 1**.

**Table 1:** Distribution of minor allele frequency of *DICER1* rs3742330[G] and *DROSHA* rs10719[A] variants in study participants.

SNP ID [minor allele]	Controls	Cases	Odds ratio (95% Confidence Interval)	*p*-value
rs3742330[G]				
POAG				
Total	0.08	0.08	0.96 (0.56–1.64)	0.87
Men	0.08	0.10	1.25 (0.62–2.52)	0.53
Women	0.08	0.05	0.63 (0.26–1.55)	0.3
PACG				
Total	0.08	0.07	0.86 (0.45–1.64)	0.63
Men	0.08	0.07	0.84 (0.33–2.17)	0.72
Women	0.08	0.07	0.86 (0.35–2.12)	0.74
rs10719[A]				
POAG				
Total	0.43	0.42	0.97 (0.73–1.29)	0.84
Men	0.45	0.42	0.88 (0.60–1.30)	0.53
Women	0.41	0.43	1.09 (0.71–1.67)	0.7
PACG				
Total	0.43	0.45	1.08 (0.78–1.48)	0.65
Men	0.45	0.38	0.75 (0.46–1.23)	0.25
Women	0.41	0.51	1.42 (0.92–2.18)	0.11

Abbreviations: POAG, primary open-angle glaucoma; PACG, primary angle-closure glaucoma.

### Genotype association analysis of rs3742330 (*DICER1*) in POAG and PACG

There is no clear inheritance pattern of the common polygenic forms of glaucoma, such as POAG and PACG. Hence, we examined the association between *DICER1* and *DROSHA* polymorphisms and the risk of POAG and PACG using SNPStat, an online software. This web tool uses different genetic models, such as co-dominant, dominant, recessive, over-dominant, and log-additive, to determine the best fit genetic model. The rs3742330 genotypes in *DICER1* showed no significant association with POAG and PACG (**Table 2**) in any of the tested genetic models. Similarly, no gender-specific genotype association was observed in POAG and PACG cases (**S1 and S2 Tables**).

**Table 2:** Genotype association analysis of rs3742330 variant in *DICER1* with primary open-angle glaucoma and primary angle-closure glaucoma.

Group	Genetic Model	Genotype	Control n (%)	Cases n (%)	Odds ratio (95% Confidence Interval)	*p*-value	*p*-value^§^
POAG	Co-dominant	A/A	204 (84.7)	130 (85.5)	1.00	0.910	0.920
		A/G	36 (14.9)	21 (13.8)	0.92 (0.51–1.64)		
		G/G	1 (0.4)	1 (0.7)	1.57 (0.10–25.31)		
	Dominant	A/A	204 (84.7)	130 (85.5)	1.00	0.810	0.770
		A/G-G/G	37 (15.3)	22 (14.5)	0.93 (0.53–1.65)		
	Recessive	A/A-A/G	240 (99.6)	151 (99.3)	1.00	0.740	0.800
		G/G	1 (0.4)	1 (0.7)	1.59 (0.10–25.60)		
	Over-dominant	A/A-G/G	205 (85.1)	131 (86.2)	1.00	0.760	0.730
		A/G	36 (14.9)	21 (13.8)	0.91 (0.51–1.63)		
	Log-additive^†^	---	---	---	0.96 (0.56–1.64)	0.870	0.820
PACG	Co-dominant	A/A	204 (84.7)	88 (86.3)	1.00	0.670	0.700
		A/G	36 (14.9)	14 (13.7)	0.90 (0.46–1.75)		
		G/G	1 (0.4)	0 (0)	0.00 (0.00-NA)		
	Dominant	A/A	204 (84.7)	88 (86.3)	1.00	0.700	0.700
		A/G-G/G	37 (15.3)	14 (13.7)	0.88 (0.45–1.70)		
	Recessive	A/A-A/G	240 (99.6)	102 (100)	1.00	0.400	0.430
		G/G	1 (0.4)	0 (0)	0.00 (0.00-NA)		
	Over-dominant	A/A-G/G	205 (85.1)	88 (86.3)	1.00	0.770	0.770
		A/G	36 (14.9)	14 (13.7)	0.91 (0.47–1.76)		
	Log-additive^†^	---	---	---	0.86 (0.45–1.64)	0.630	0.640

^†^Additive model also non-significant

^§^p-value adjusted for age and sex in the overall group and by age in men and women groups.

Abbreviations: POAG, primary open-angle glaucoma PACG, primary angle-closure glaucoma.

### Genotype association analysis of rs10719 (*DROSHA*) in POAG and PACG

No significant genotype association of rs10719 was observed in POAG and PACG cases compared to controls in the co-dominant, dominant, over-dominant, recessive, and log-additive genetic models (**Table 3**). Likewise, the gender-stratified analysis showed no significant genotype distribution in POAG and PACG (**S3 and S4 Tables**).

**Table 3:** Genotype association analysis of rs10719 variant in *DROSHA* with primary open-angle glaucoma and primary angle-closure glaucoma.

Group	Genetic Model	Genotype	Control n (%)	Cases n (%)	Odds ratio (95% Confidence Interval)	*p*-value	*p*-value^§^
POAG	Co-dominant	G/G	82 (33.3)	49 (32.7)	1.00	0.820	0.840
		A/G	116 (47.1)	75 (50)	1.08 (0.68–1.71)		
		A/A	48 (19.5)	26 (17.3)	0.91 (0.50–1.64)		
	Dominant	G/G	82 (33.3)	49 (32.7)	1.00	0.890	0.920
		A/G-A/A	164 (66.7)	101 (67.3)	1.03 (0.67–1.59)		
	Recessive	G/G-A/G	198 (80.5)	124 (82.7)	1.00	0.590	0.610
		A/A	48 (19.5)	26 (17.3)	0.86 (0.51–1.47)		
	Over-dominant	G/G-A/A	130 (52.9)	75 (50.0)	1.00	0.580	0.620
		A/G	116 (47.1)	75 (50.0)	1.12 (0.75–1.68)		
	Log-additive^†^	---	---	---	0.97 (0.73–1.29)	0.840	0.830
PACG	Co-dominant	G/G	82 (33.3)	34 (33.7)	1.00	0.620	0.630
		A/G	116 (47.1)	43 (42.6)	0.89 (0.53–1.52)		
		A/A	48 (19.5)	24 (23.8)	1.21 (0.64–2.27)		
	Dominant	G/G	82 (33.3)	34 (33.7)	1.00	0.950	0.900
		A/G-A/A	164 (66.7)	67 (66.3)	0.99 (0.60–1.61)		
	Recessive	G/G-A/G	198 (80.5)	77 (76.2)	1.00	0.380	0.410
		A/A	48 (19.5)	24 (23.8)	1.29 (0.74–2.24)		
	Over-dominant	G/G-A/A	130 (52.9)	58 (57.4)	1.00	0.440	0.420
		A/G	116 (47.1)	43 (42.6)	0.83 (0.52–1.33)		
	Log-additive^†^	---	---	---	1.08 (0.78–1.48)	0.650	0.700

^†^Additive model also non-significant; ^§^p-value adjusted for age and sex in the overall group and by age in men and women groups.

Abbreviations: POAG, primary open-angle glaucoma, PACG, primary angle-closure glaucoma.

### Regression analysis and genotype influence on clinical parameters

**Fig 2:**
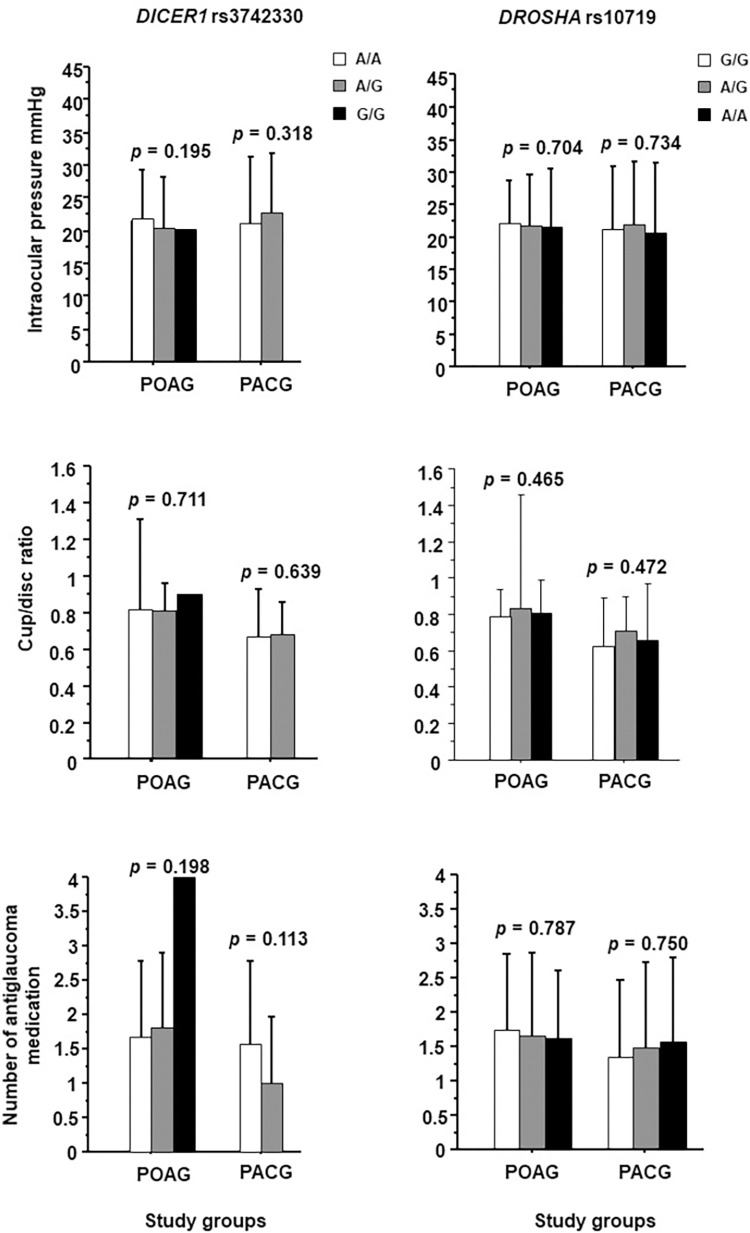
Genotype effects of rs3742330 in *DICER1* and rs10719 in *DROSHA* on intraocular pressure, cup/disc ratio, and the number of antiglaucoma medications in POAG and PACG patient groups. The bar graphs represent the mean ± standard deviation for intraocular pressure, cup/disc ratio, and the number of antiglaucoma medications. The *p*-value shown is calculated by the Kruskal-Wallis H test for three genotype groups and the Mann-Whitney U-test for the two genotype groups observed for the rs3742330 polymorphisms in the PACG group, where no homozygous G/G genotype was observed.

A binary regression analysis was performed to assess the influence of multiple risk factors like age, sex, and genotypes of rs3742330 and rs10719 on POAG/PACG outcome. None of the variables significantly contributed to the risk of POAG and PACG (**Table 4**). In addition, both the polymorphisms rs3742330 (*DICER1*) and rs10719 (*DROSHA*) showed no significant genotype influence on glaucoma-specific clinical indices such as IOP, cup/disk ratio, and the number of antiglaucoma medications (**Fig 2**).

**Table 4:** Binary logistic regression analysis to determine the effect of age, sex, and polymorphisms on glaucoma risk.

Group Variables	B	SE	Wald	Odds ratio (95% Confidence Interval)	*p*-value
**POAG**					
Age	0.016	0.012	1.704	1.01 (0.99–1.04)	0.192
Sex	0.076	0.210	0.130	1.08 (0.71–1.63)	0.719
Rs3742330			0.243		0.886
A/G	-0.123	0.303	0.165	0.88 (0.49–1.60)	0.685
G/G	0.384	1.426	0.072	1.46 (0.09–24.00)	0.788
Rs10719			0.286		0.867
A/G	0.085	0.236	0.130	1.09 (0.68–1.73)	0.718
A/A	-0.056	0.307	0.034	0.94 (0.51–1.72)	0.855
Constant	-1.495	0.760	3.870	0.22	0.049
**PACG**					
Age	0.021	0.016	1.817	1.02 (0.99–1.05)	0.178
Sex	-0.337	0.241	1.966	0.71 (0.44–1.14)	0.161
Rs3742330			0.098		0.952
A/G	-0.108	0.343	0.098	0.898 (0.45–1.76)	0.754
G/G	-20.473	40192.970	0.000	0 (0)	1.000
Rs10719			1.234		0.540
A/G	-0.131	0.274	0.228	0.87 (0.51–1.50)	0.633
A/A	0.217	0.328	0.439	1.24 (0.65–2.36)	0.508
Constant	-1.952	0.966	4.086	0.142	0.043

Abbreviations: POAG, primary open-angle glaucoma; PACG, primary angle-closure glaucoma.

### Combined allelic association analysis of rs3742330 and rs10719 with POAG and PACG

In order further examine the association of variants rs3742330 (A>G) and rs10719 (A>G) in POAG and PACG, a combined allelic association analysis was performed using the SHEsis online software. However, none of the allelic combinations of the two variants showed any significant association with POAG and PACG, as shown in Table 5.

**Table 5:** Combined allelic association analysis in POAG and PACG.

Allele combination*	Cases (Frequency)	Control (Frequency)	Chi^2^	Fisher’s *p*	Odds Ratio (95% Confidence Interval)
**POAG** ^†^					
A-G	0.51	0.53	0.198	0.656	0.93 (0.70~1.25)
A-A	0.40	0.38	0.352	0.553	1.09 (0.81~1.46)
G-G	0.06	0.04	1.317	0.251	1.48 (0.75~2.92)
G-A	0.02	0.04	3.054	0.080	0.43 (0.16~1.13)
**PACG** ^‡^					
A-G	0.51	0.53	0.256	0.612	0.92 (0.66~1.27)
A-A	0.41	0.38	0.561	0.453	1.13 (0.81~1.58)
G-G	0.04	0.04	0.014	0.904	0.95 (0.40~2.26)
G-A	0.03	0.04	0.225	0.635	0.80 (0.32~1.97)

* In the order of rs3742330 and rs10719.

^†^ Global Chi^2^ = 4.52, df = 3, Fisher’s *p*-value is 0.210.

^‡^ Global Chi^2^ = 0.69,df = 3, Fisher’s *p*-value = 0.875.

## Discussion

Genome-wide association studies have previously investigated the role of different genes in POAG and PACG and identified several genetic loci that account for a small percentage of the disease or are associated with specific ethnic groups [9]. Although substantial molecular insights have been gained through these studies, further studies are still warranted to identify other genetic variants that may be linked to the development or progression of the disease. Although studies have reported the genetic association of miRNA biogenesis gene polymorphisms, *DICER1* (rs3642330) and *DROSHA* (rs10719), in a wide variety of human diseases [41,48–51], however, their genetic contribution in glaucoma has not been much investigated. In this study, we report no association of polymorphisms (rs3742330 and rs10719) in genes (*DICER1* and *DROSHA*, respectively) involved in miRNA biogenesis in the POAG and PACG Middle-Eastern cohort of Saudi Arab ethnicity.

The MAF of rs3742330(G) in *DICER1* was 0.08, 0.07, and 0.08 in POAG, PACG, and control samples of Saudi ethnicity, respectively, which were not statistically significant. As compared to the NCBI database (https://www.ncbi.nlm.nih.gov/snp/rs3742330), the allele frequency was similar to that of the Europeans (0.09), lower than Asians (0.36) but higher than Africans (0.028) and African Americans (0.029). According to the NCBI (https://www.ncbi.nlm.nih.gov/snp/rs10719), rs10719[A] is a reference and a major allele in Africans, East Asians, and Japanese but is a minor allele in Europeans, South Asians, and Americans as observed in our study cohort. Accordingly, the MAF of rs10719(A) in *DROSHA* was 0.42, 0.45, and 0.43 among POAG, PACG, and controls of this ethnic group, respectively, which were non-significant. In comparison to the NCBI database, the allele frequency was lower than Asians (0.69), Africans (0.63), and African Americans (0.63), but higher than Europeans (0.20), South Asians (0.39), and Americans (0.34). The allele frequency distribution indicates the ethnic variability of these polymorphisms across different populations.

The catalytic role of DROSHA and DICER in synthesizing miRNAs is central to RNA-mediated gene silencing or RNA interference [19]. Deletion of *DROSHA* and *DICER* in human cell lines completely abolished or markedly reduced miRNA production in the canonical pathway [52]. Emerging evidence suggests that polymorphism(s) in these genes (*DICER1 and DROSHA)* may alter the biological functions of miRNAs and contribute to the pathogenesis of various systemic and neurodegenerative diseases [37–39,53,54]. miRNAs exhibit tissue-specific expression and are known to express in glaucoma-related ocular tissues [55]. Several studies have highlighted the significant functional role of miRNAs in glaucoma [56]. Also, miRNA-related variants are reported to be associated with POAG endophenotypes [57]. Although there are no published reports of these polymorphisms (rs3742330 and rs10719) being examined in glaucoma types, Chatzikyriakidou et al. reported a different *DICER1* variant, rs1057035 (C>T), which conferred protection (OR of 0.69) in patients with pseudoexfoliation syndrome [40] but was not associated with POAG. Using the LDlink analysis (https://ldlink.nci.nih.gov/?tab=ldpop), we have previously examined the linkage between rs1057035 and rs3742330 in *DICER1* across the 1000 Genomes database and noted no linkage between these two SNPs (r^2^ = 0.032) [43]. Nonetheless, the data further support the absence of any association of the *DICER1* variant with POAG observed in our patient cohort. There are no published reports of variant(s) in these genes investigated in PACG.

DROSHA initiates miRNA maturation in the nucleus by recognizing and cleaving hairpin precursors embedded in primary transcripts [58]. Accumulating evidence suggests a molecular role of DROSHA in the regulation of diverse aspects of RNA metabolism, protection against genotoxic stresses and potentially harmful elements at the cellular level, and regulation of cell fate determination and timely differentiation at the physiological level [58]. However, beyond miRNA biogenesis, little is known about DROSHA regulation in pathological conditions. Yang et al. revealed that p38 MAPK directly phosphorylates and triggers degradation of DROSHA under stress conditions and inhibits cell survival [59]. The same group also showed that 6-hydroxydopamine (6-OHDA), a neurotoxin associated with Parkinson’s disease, destabilized DROSHA via p38 MAPK phosphorylation in a mouse model of Parkinson’s disease. Interestingly, restoring the DROSHA level protected the dopamine-releasing neurons and improved the motor deficits, highlighting DROSHA’s role in survival of dopaminergic neurons [41].

*DICER* is essential in development and angiogenesis [60,61]. Carriers of *DICER1* mutants are reported to have optic nerve damage and retinal abnormalities [62]. Donor human eyes with geographic atrophy, a form of age-related macular degeneration, was reported to have reduced *DICER1* mRNA in macular RPE [63]. Conditional ablation of *Dicer1* was demonstrated to induce Alu RNA accumulation in human and mouse RPE cells leading to RPE cytotoxicity and degeneration. These findings revealed a miRNA-independent role of *DICER1* in cell survival [63]. Inactivation of *Dicer* in the mouse retina can cause retinal degeneration [64]. Aberrant *DROSHA* and *DICER1* expression are associated with various types and stages of cancers, albeit with inconsistent findings [65–68]. The underlying potential mechanism(s) linking *DROSHA* and *DICER1* polymorphisms (rs10719 and rs3742330) to glaucoma pathogenesis is unknown. Plausibly, altered levels of DROSHA and DICER1 (as a result of these polymorphisms) may thus either directly affect enzyme function or indirectly, via differential regulation of miRNA expression profiles, can influence disease pathogenesis as discussed above. Rs3742330 polymorphism has been related to *DICER1* mRNA dysregulation, wherein the polymorphic A/G and G/G genotypes harbored lower levels of *DICER1* mRNA [31,69]. However, the polymorphisms rs3742330 in *DICER1* and rs10719 in *DROSHA* were not associated with POAG and PACG or its related clinical endophenotypes (e.g., IOP and cup/disc ratio) in our Saudi cohort.

POAG and PACG are genetically complex traits with multiple genetic variants implicated in their etiology. Due to their complex pattern of inheritance, the molecular mechanism(s) of the disease could highly likely involve the epistatic interaction of gene-gene and gene-environment factors. Each of these could have a relatively small effect and low penetrance, but may still contribute to many cases in a population. However, analysis of combined allelic haplotypes of rs3742330 and rs10719 showed no significant association with POAG and PACG.

The study has certain limitations. The study is limited by sample size, with no mechanistic evidence. Only a single variant in these genes was investigated in a group of patients of a specific ethnicity. Since our hospital is a tertiary care center, there may be a potential sample bias. Though the sample size was limited, based on the observed allele frequencies, our study had an estimated power of >0.8 per allele for rs3742330 (*DICER1*) and >0.9 per allele for the rs10719 (*DROSHA*) variant to detect an odds risk of 2.0 with an alpha type I error of 5%. Nonetheless, it needs investigation in a much larger population-based sample size to detect an odds risk ≤1.5, as is commonly observed in genetic association studies of complex polygenic diseases.

In conclusion, our study revealed that the 3’ UTR polymorphisms, rs3742330 in *DICER1* and rs10719 in *DROSHA*, are neither associated with POAG and PACG nor the clinical indices of glaucoma, such as IOP, cup/disc ratio, and the number of antiglaucoma medications in the Middle-Eastern cohort of Saudi Arab ethnicity. This is the first study to investigate and report no association of these polymorphisms in POAG and PACG patients in Arabs of Saudi origin. The findings of this study add to the genetic basis of glaucoma literature in POAG and PACG in this ethnicity. However, the sample size of this study is relatively small (but sufficient for an exploratory analysis using a candidate gene approach). But we cannot completely rule out the role of different polymorphisms in these or other genes (e.g., *DGCR8*, *XPO5*) significant to the miRNA biogenesis pathway in glaucoma. Hence, the findings require additional molecular epidemiological studies for further confirmation in a more comprehensive population-based sample, probably with age and sex-matched control groups and in other ethnicities.

## Supporting information

S1 Fig The genomic region containing (A) rs3742330 in DICER1 and (B) rs10719 in DROSHA and its neighboring features as annotated from UCSC browser and TargetScanHuman v7.0.
(PDF)

S2 Fig Schematic representation of the regulation of miRNA biogenesis by DICER1 and DROSHA. (PDF)

S3 Fig Representative image of visual field defect with HVF, 24–2 strategy in (A) POAG and (B) PACG patient.
(PDF)

S1 Table Genotype association analysis of rs3742330 variant in DICER1 with primary open-angle glaucoma according to gender. (PDF)

S2 Table Genotype association analysis of rs3742330 variant in DICER1 with primary angle-closure glaucoma according to gender. (PDF)

S3 Table Genotype association analysis of rs10719 variant in DROSHA with primary open-angle glaucoma according to gender. (PDF)

S4 Table Association analysis of rs10719 variant in DROSHA with primary angle-closure glaucoma according to gender. (PDF)
