# Supplementary material for: The 3’ UTR polymorphisms rs3742330 in DICER1 and rs10719 in DROSHA genes are not associated with primary open-angle and angle-closure glaucoma: As case-control study
Source: PLoS One. 2023 Apr 26;18(4):e0284852. doi: 10.1371/journal.pone.0284852 (PMC10132650; doi:10.1371/journal.pone.0284852)
Supplement: S1 Fig — The genomic region containing (A) rs3742330 in DICER1 and (B) rs10719 in DROSHA and its neighboring features as annotated from UCSC browser and TargetScanHuman v7.0. (PDF) [file pone.0284852.s001.pdf]

S1 Fig. The genomic region containing (A) rs3742330 in *DICER1* and (B) rs10719 in *DROSHA* and its neighboring features as annotated from UCSC browser and TargetScanHuman v7.0. It can be noted that the gene in expressed in many tissues and conserved in multiple species. Also, the polymorphic region is close to multiple miRNA binding sites, transcript factors, and histone modification sites suggesting that these genes might have a regulatory role.

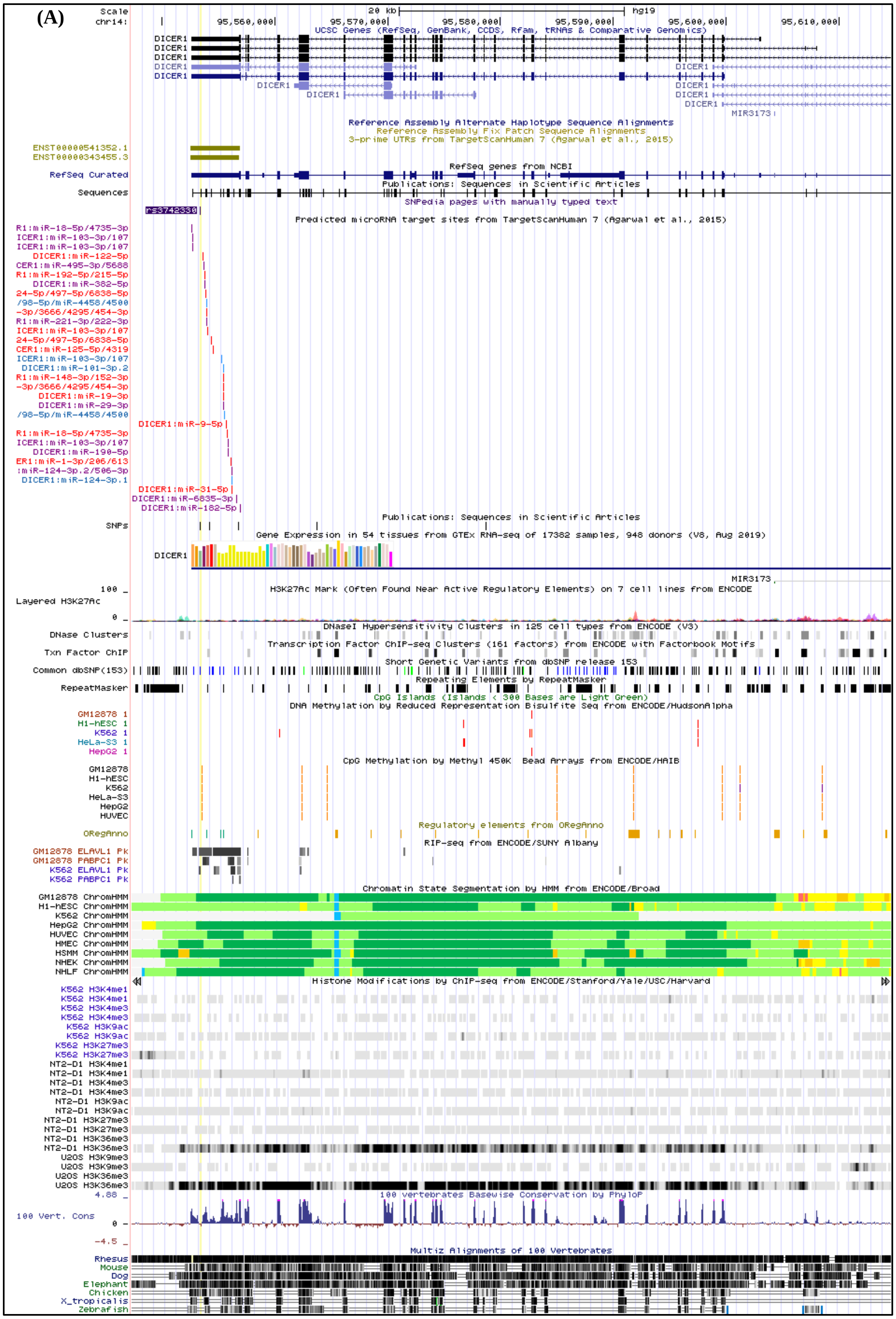

(B)

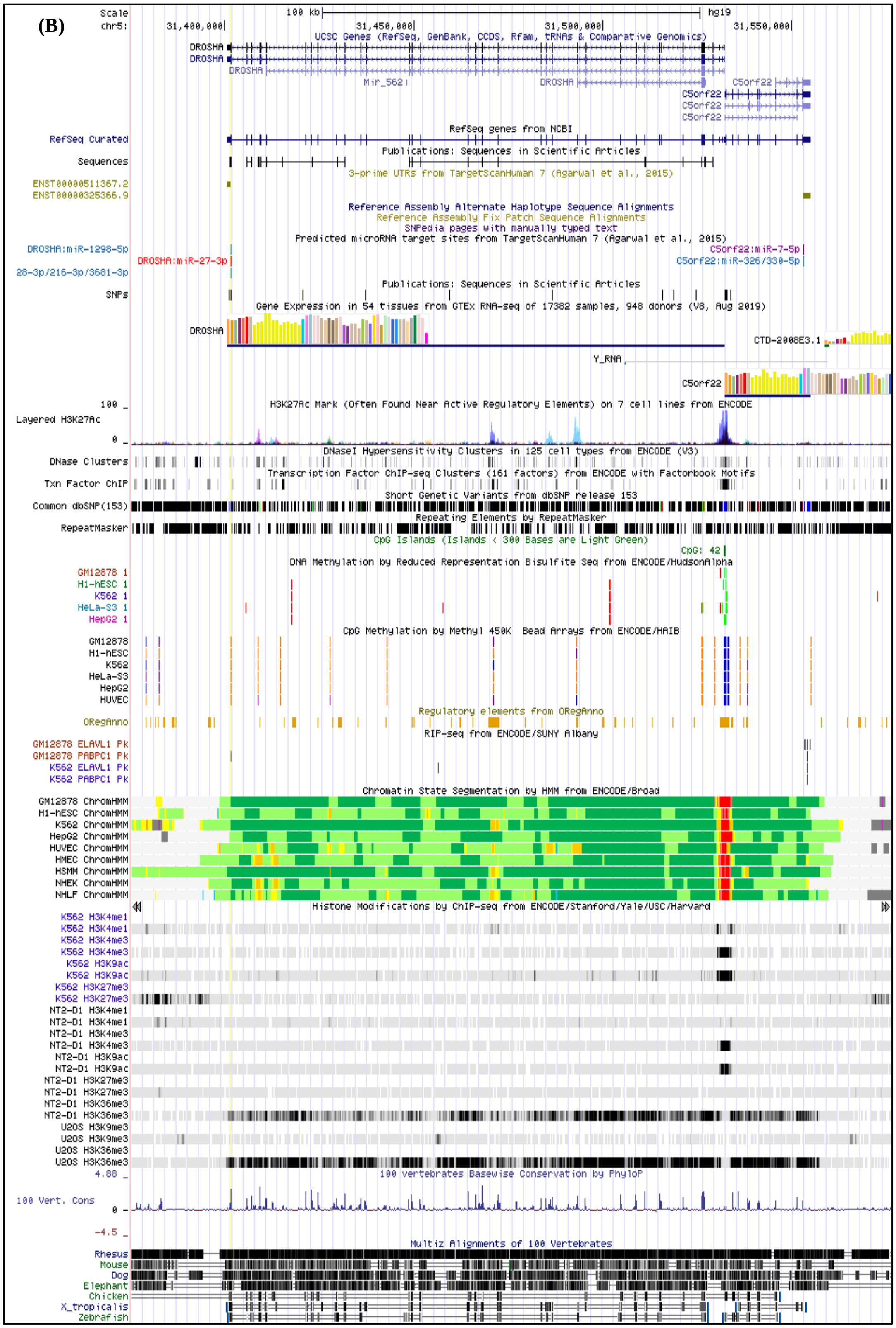

Scale
chr5:
100 kb
hg19
31,400,000
31,450,000
31,500,000
31,550,000
UCSC Genes (RefSeq, GenBank, CCDS, Rfam, tRNAs & Comparative Genomics)
DROSHA
Mir_562
C5orf22
RefSeq genes from NCBI
RefSeq Curated
Sequences
Publications: Sequences in Scientific Articles
3-prime UTRs from TargetScanHuman 7 (Agarwal et al., 2015)
ENST00000511367.2
ENST00000325366.9
Reference Assembly Alternate Haplotype Sequence Alignments
Reference Assembly Fix Patch Sequence Alignments
SNPedia pages with manually typed text
Predicted microRNA target sites from TargetScanHuman 7 (Agarwal et al., 2015)
DROSHA:miR-1298-5p
DROSHA:miR-27-3p
28-3p/216-3p/3681-3p
C5orf22:miR-7-5p
C5orf22:miR-326/330-5p
SNPs
Gene Expression in 54 tissues from GTEx RNA-seq of 17382 samples, 948 donors (V8, Aug 2019)
CTD-2008E3.1
Y_RNA
100
Layered H3K27Ac
0
H3K27Ac Mark (Often Found Near Active Regulatory Elements) on 7 cell lines from ENCODE
DNaseI Hypersensitivity Clusters in 125 cell types from ENCODE (V3)
DNase Clusters
Transcription Factor ChIP-seq Clusters (161 factors) from ENCODE with Factorbook Motifs
Txn Factor ChIP
Short Genetic Variants from dbSNP release 153
Common dbSNP(153)
Repeating Elements by RepeatMasker
RepeatMasker
CpG Islands (Islands < 300 Bases are Light Green)
CpG: 42
DNA Methylation by Reduced Representation Bisulfite Seq from ENCODE/HudsonAlpha
GM12878 1
H1-hESC 1
K562 1
HeLa-S3 1
HepG2 1
CpG Methylation by Methyl 450K Bead Arrays from ENCODE/HAIB
GM12878
H1-hESC
K562
HeLa-S3
HepG2
HUVEC
Regulatory elements from ORegAnno
ORegAnno
RIP-seq from ENCODE/SUNY Albany
GM12878 ELAVL1 Pk
GM12878 PABPC1 Pk
K562 ELAVL1 Pk
K562 PABPC1 Pk
Chromatin State Segmentation by HMM from ENCODE/Broad
GM12878 ChromHMM
H1-hESC ChromHMM
K562 ChromHMM
HepG2 ChromHMM
HUVEC ChromHMM
HMEC ChromHMM
HSMM ChromHMM
NHEK ChromHMM
NHLF ChromHMM
Histone Modifications by ChIP-seq from ENCODE/Stanford/Yale/USC/Harvard
K562 H3K4me1
K562 H3K4me1
K562 H3K4me3
K562 H3K4me3
K562 H3K9ac
K562 H3K9ac
K562 H3K27me3
K562 H3K27me3
NT2-D1 H3K4me1
NT2-D1 H3K4me1
NT2-D1 H3K4me3
NT2-D1 H3K4me3
NT2-D1 H3K9ac
NT2-D1 H3K9ac
NT2-D1 H3K27me3
NT2-D1 H3K27me3
NT2-D1 H3K36me3
NT2-D1 H3K36me3
U2OS H3K9me3
U2OS H3K9me3
U2OS H3K36me3
U2OS H3K36me3
4.88
100 vertebrates Basewise Conservation by PhyloP
100 Vert. Cons
0
-4.5
Multiz Alignments of 100 Vertebrates
Rhesus
Mouse
Dog
Elephant
Chicken
X_tropicalis
Zebrafish
