# Supplementary material for: The 3’ UTR polymorphisms rs3742330 in DICER1 and rs10719 in DROSHA genes are not associated with primary open-angle and angle-closure glaucoma: As case-control study
Source: PLoS One. 2023 Apr 26;18(4):e0284852. doi: 10.1371/journal.pone.0284852 (PMC10132650; doi:10.1371/journal.pone.0284852)

S2 Fig. Schematic representation of the regulation of miRNA biogenesis by DICER1 and DROSHA. The presence of *DICER1* rs3742330 and/or *DROSHA* rs10719 polymorphisms located in the 3′-UTR might inhibit or downregulate the function of these miRNA processing proteins by impacting the binding site of their miRNAs, resulting in dysregulation in processing of miRNA related gene/protein expression and impact downstream cellular processes and pathways to influence disease outcomes.

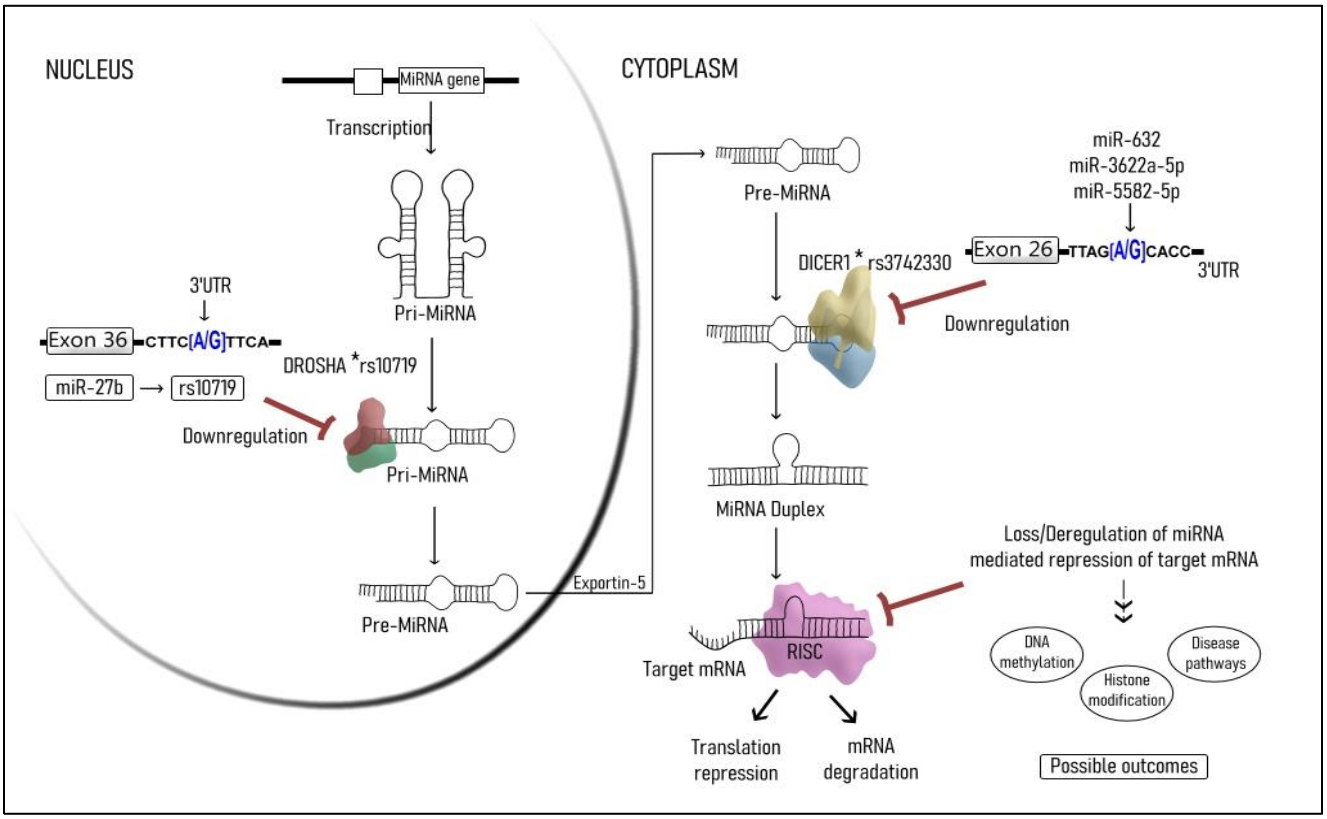

Supplement: S2 Fig — (PDF) [file pone.0284852.s002.pdf]
