# Supplementary material for: The 3’ UTR polymorphisms rs3742330 in DICER1 and rs10719 in DROSHA genes are not associated with primary open-angle and angle-closure glaucoma: As case-control study
Source: PLoS One. 2023 Apr 26;18(4):e0284852. doi: 10.1371/journal.pone.0284852 (PMC10132650; doi:10.1371/journal.pone.0284852)

S3 Fig. Representative image of visual field defect with HVF, 24-2 strategy in (A) POAG and (B) PACG patient.

(A)

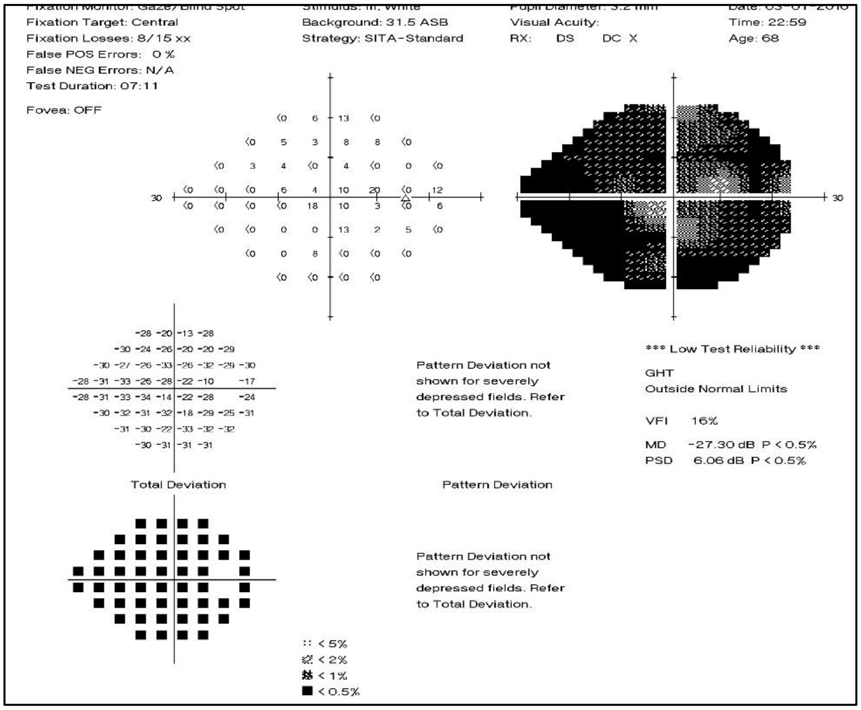

(B)

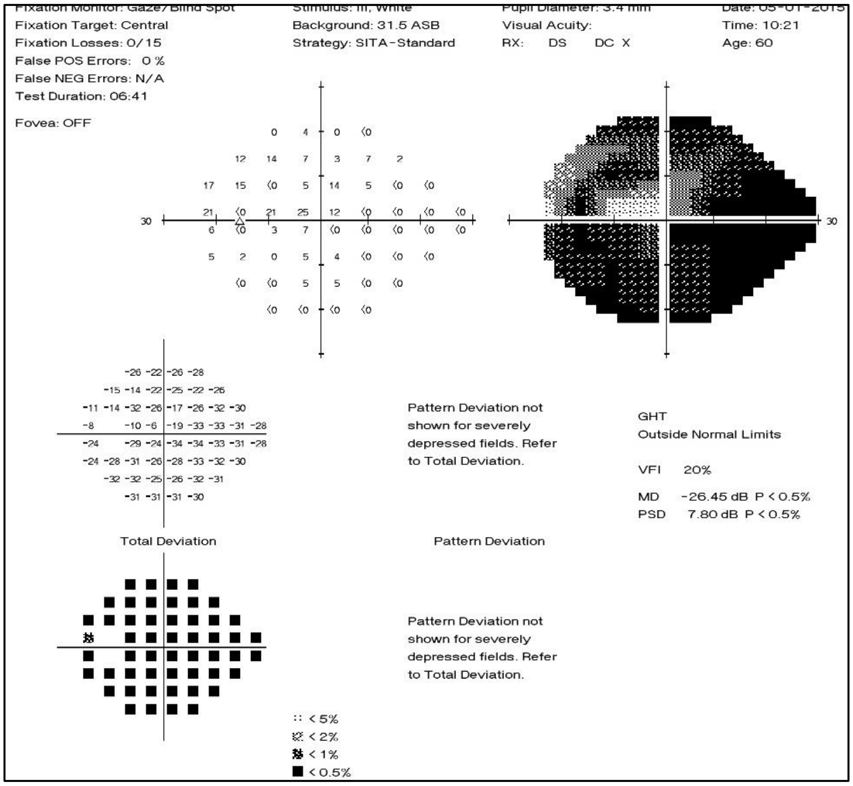

Supplement: S3 Fig — Representative image of visual field defect with HVF, 24–2 strategy in (A) POAG and (B) PACG patient. (PDF) [file pone.0284852.s003.pdf]
