# Supplementary material for: The 3’ UTR polymorphisms rs3742330 in DICER1 and rs10719 in DROSHA genes are not associated with primary open-angle and angle-closure glaucoma: As case-control study
Source: PLoS One. 2023 Apr 26;18(4):e0284852. doi: 10.1371/journal.pone.0284852 (PMC10132650; doi:10.1371/journal.pone.0284852)
Supplement: S1 Table — (PDF) [file pone.0284852.s004.pdf]

S1 Table: Genotype association analysis of rs3742330 variant in *DICER1* with primary open-angle glaucoma according to gender

Group	Genetic Model	Genotype	Control n (%)	POAG n (%)	Odds ratio (95% Confidence Interval)	*p*-value	*p*-value§
Men	Co-dominant	A/A	109 (85.2)	68 (81.0)	1.00		
		A/G	18 (14.1)	16 (19.1)	1.42 (0.68-2.98)	0.390	0.390
		G/G	1 (0.8)	0 (0)	0.00 (0.00-NA)		
	Dominant	A/A	109 (85.2)	68 (81.0)	1.00	0.420	0.430
		A/G-G/G	19 (14.8)	16 (19.1)	1.35 (0.65-2.80)		
	Recessive	A/A-A/G	127 (99.2)	84 (100)	1.00	0.310	0.310
		G/G	1 (0.8)	0 (0)	0.00 (0.00-NA)		
	Over-dominant	A/A-G/G	110 (85.9)	68 (81.0)	1.00	0.340	0.340
		A/G	18 (14.1)	16 (19.1)	1.44 (0.69-3.01)		
	Log-additive†	---	---	---	1.25 (0.62-2.52)	0.530	0.540
Women	Co-dominant	A/A	95 (84.1)	62 (91.2)	1.00		
		A/G	18 (15.9)	5 (7.3)	0.43 (0.15-1.21)	0.088	0.100
		G/G	0 (0)	1 (1.5)	NA (0.00-NA)		
	Dominant	A/A	95 (84.1)	62 (91.2)	1.00	0.160	0.150
		A/G-G/G	18 (15.9)	6 (8.8	0.51 (0.19-1.36)		
	Recessive	A/A-A/G	113 (100.0)	67 (98.5)	1.00	0.160	0.200
		G/G	0 (0)	1 (1.5)	NA (0.00-NA)		
	Over-dominant	A/A-G/G	95 (84.1)	63 (92.7)	1.00	0.0820	0.084
		A/G	18 (15.9)	5 (7.3)	0.42 (0.15-1.19)		
	Log-additive†	---	---	---	0.63 (0.26-1.55)	0.300	0.280

†Additive model also non-significant; §p-value adjusted for age and sex in overall group and by age in men and women groups
Abbreviations: POAG, primary open-angle glaucoma BIC, Bayesian information criterion
