# Supplementary material for: The 3’ UTR polymorphisms rs3742330 in DICER1 and rs10719 in DROSHA genes are not associated with primary open-angle and angle-closure glaucoma: As case-control study
Source: PLoS One. 2023 Apr 26;18(4):e0284852. doi: 10.1371/journal.pone.0284852 (PMC10132650; doi:10.1371/journal.pone.0284852)
Supplement: S2 Table — (PDF) [file pone.0284852.s005.pdf]

S2 Table: Genotype association analysis of rs3742330 variant in *DICER1* with primary angle-closure glaucoma according to gender

Group	Genetic Model	Genotype	Control n (%)	PACG n (%)	Odds ratio (95% Confidence Interval)	*p*-value	*p*-value§
Men	Co-dominant	A/A	109 (85.2)	39 (86.7)	1.00		
		A/G	18 (14.1)	6 (13.3)	0.93 (0.34-2.52)	0.730	0.730
		G/G	1 (0.8)	0 (0)	0.00 (0.00-NA)		
	Dominant	A/A	109 (85.2)	39 (86.7)	1.00	0.800	0.850
		A/G-G/G	19 (14.8)	6 (13.3)	0.88 (0.33-2.37)		
	Recessive	A/A-A/G	127 (99.2)	45 (100)	1.00	0.440	0.430
		G/G	1 (0.8)	0 (0%)	0.00 (0.00-NA)		
	Over-dominant	A/A-G/G	110 (85.9)	39 (86.7)	1.00	0.900	0.950
		A/G	18 (14.1)	6 (13.3)	0.94 (0.35-2.54)		
	Log-additive†	---	---	---	0.84 (0.33-2.17)	0.720	0.760
Women	--	A/A	95 (84.1)	49 (86.0)	1.00		
		A/G	18 (15.9)	8 (14.0)	0.86 (0.35-2.12)	0.740	0.710
		G/G	0 (0)	0 (0)	--		

†Additive model also non-significant; §p-value adjusted for age and sex in overall group and by age in men and women groups
Abbreviations: PACG, primary angle-closure glaucoma
