# Supplementary material for: The 3’ UTR polymorphisms rs3742330 in DICER1 and rs10719 in DROSHA genes are not associated with primary open-angle and angle-closure glaucoma: As case-control study
Source: PLoS One. 2023 Apr 26;18(4):e0284852. doi: 10.1371/journal.pone.0284852 (PMC10132650; doi:10.1371/journal.pone.0284852)
Supplement: S3 Table — (PDF) [file pone.0284852.s006.pdf]

S3 Table: Genotype association analysis of rs10719 variant in *DROSHA* with primary open-angle glaucoma according to gender

Group	Genetic Model	Genotype	Control n (%)	POAG n (%)	Odds ratio (95% Confidence Interval)	*p*-value	*p*-value§
Men	Co-dominant	G/G	40 (30.3)	30 (36.1)	1.00	0.650	0.660
		A/G	66 (50)	37 (44.6)	0.75 (0.40-1.39)		
		A/A	26 (19.7)	16 (19.3)	0.82 (0.38-1.79)		
	Dominant	G/G	40 (30.3)	30 (36.1)	1.00	0.370	0.380
		A/G-A/A	92 (69.7)	53 (63.9)	0.77 (0.43-1.37)		
	Recessive	G/G-A/G	106 (80.3)	67 (80.7)	1.00	0.940	0.940
		A/A	26 (19.7)	16 (19.3)	0.97 (0.49-1.95)		
	Over-dominant	G/G-A/A	66 (50.0)	46 (55.4)	1.00	0.440	0.440
		A/G	66 (50.0)	37 (44.6)	0.80 (0.46-1.40)		
	Log-additive†	---	---	---	0.88 (0.60-1.30)	0.530	0.540
Women	Co-dominant	G/G	42 (36.8)	19 (28.4)	1.00	0.250	0.310
		A/G	50 (43.9)	38 (56.7)	1.68 (0.85-3.34)		
		A/A	22 (19.3)	10 (14.9)	1.00 (0.40-2.53)		
	Dominant	G/G	42 (36.8)	19 (28.4)	1.00	0.240	0.290
		A/G-A/A	72 (63.2)	48 (71.6)	1.47 (0.77-2.83)		
	Recessive	G/G-A/G	92 (80.7)	57 (85.1)	1.00	0.450	0.490
		A/A	22 (19.3)	10 (14.9)	0.73 (0.32-1.66)		
	Over-dominant	G/G-A/A	64 (56.1)	29 (43.3)	1.00	0.094	0.130
		A/G	50 (43.9)	38 (56.7)	1.68 (0.91-3.08)		
	Log-additive†	---	---	---	1.09 (0.71-1.67)	0.700	0.730

†Additive model also non-significant; §p-value adjusted for age and sex in overall group and by age in men and women groups
Abbreviations: POAG, primary open-angle glaucoma.
