# Supplementary material for: The 3’ UTR polymorphisms rs3742330 in DICER1 and rs10719 in DROSHA genes are not associated with primary open-angle and angle-closure glaucoma: As case-control study
Source: PLoS One. 2023 Apr 26;18(4):e0284852. doi: 10.1371/journal.pone.0284852 (PMC10132650; doi:10.1371/journal.pone.0284852)
Supplement: S4 Table — (PDF) [file pone.0284852.s007.pdf]

S4 Table: Association analysis of rs10719 variant in *DROSHA* with primary angle-closure glaucoma according to gender

Group	Genetic Model	Genotype	Control n (%)	PACG n (%)	Odds ratio (95% Confidence Interval)	*p*-value	*p*-value§
Men	Co-dominant	G/G	40 (30.3)	18 (40.0)	1.00	0.480	0.460
		A/G	66 (50.0)	20 (44.4)	0.67 (0.32-1.42)		
		A/A	26 (19.7)	7 (15.6)	0.60 (0.22-1.63)		
	Dominant	G/G	40 (30.3)	18 (40.0)	1.00	0.240	0.210
		A/G-A/A	92 (69.7)	27 (60.0)	0.65 (0.32-1.32)		
	Recessive	G/G-A/G	106 (80.3)	38 (84.4)	1.00	0.530	0.570
		A/A	26 (19.7)	7 (15.6)	0.75 (0.30-1.87)		
	Over-dominant	G/G-A/A	66 (50.0)	25 (55.6)	1.00	0.520	0.450
		A/G	66 (50.0)	20 (44.4)	0.80 (0.41-1.58)		
	Log-additive†	---	---	---	0.75 (0.46-1.23)	0.250	0.250
Women	Co-dominant	G/G	42 (36.8)	16 (28.6)	1.00	0.250	0.270
		A/G	50 (43.9)	23 (41.1)	1.21 (0.57-2.58)		
		A/A	22 (19.3)	17 (30.4)	2.03 (0.86-4.77)		
	Dominant	G/G	42 (36.8)	16 (28.6)	1.00	0.280	0.310
		A/G-A/A	72 (63.2)	40 (71.4)	1.46 (0.73-2.92)		
	Recessive	G/G-A/G	92 (80.7)	39 (69.6)	1.00	0.110	0.120
		A/A	22 (19.3)	17 (30.4)	1.82 (0.87-3.80)		
	Over-dominant	G/G-A/A	64 (56.1)	33 (58.9)	1.00	0.730	0.710
		A/G	50 (43.9)	23 (41.1)	0.89 (0.47-1.71)		
	Log-additive†	---	---	---	1.42 (0.92-2.18)	0.110	0.130

†Additive model also non-significant; §p-value adjusted for age and sex in overall group and by age in men and women groups
Abbreviations: PACG, primary angle-closure glaucoma
